# Urinary Cadmium as a Marker of Exposure in Epidemiological Studies: Bernard and Chaumont Respond

**DOI:** 10.1289/ehp.1307376R

**Published:** 2013-10-01

**Authors:** Alfred Bernard, Agnès Chaumont

**Affiliations:** Laboratory of Toxicology and Applied Pharmacology, Catholic University of Louvain, Brussels, Belgium

We thank Adams and Newcomb for their interest in our article about the significance of low-level urinary cadmium (U-Cd) (Chaumont et al. 2013). Adams and Newcomb are correct that a constant offset between curves on a log scale implies a constant ratio but not a difference. The issue, however, is that the U-Cd ratio between current smokers and never-smokers in Figure 2 of our article does not show the increase seen in the ratio of kidney Cd concentrations because the concentration was already increased at 20 years of age (~ 1.40), peaked around 40 years (~ 1.80), and returned to its baseline level at 60 years (~ 1.50) and even lower at 70 years (~ 1.30). Although U-Cd steadily increased with age, the increase of U-Cd in current smokers leveled off around 40 years of age. Thus, at the ages of 20, 30, 40, 60, and 70 years, the differences in U-Cd between current smokers and never-smokers were 0.09, 0.16, 0.29, 0.29, and 0.20 µg/L, respectively. The increase in U-Cd in smokers leveled off around 30 years of age, evident when U-Cd curves were fitted on a linear scale (0.12, 0.25, 0.33, 0.29, and 0.21 µg/L for 20, 30, 40, 60, and 70 years, respectively). This is not the expected result for a biomarker that is envisaged to reflect the rise of Cd body burden in smokers.

Adams and Newcomb also cite several studies that have demonstrated higher U-Cd in former smokers. We will not comment on the study by Adams and Newcomb (2013), which was not yet available at the time this letter was written, nor on the study of Gunier et al. (2013), which actually did not compare U-Cd between former and current smokers. McElroy et al. (2007) and Adams et al. (2011) reported higher creatinine-adjusted U-Cd in women who were former smokers compared with those who never smoked. However, it would be interesting to consolidate these findings by analyzing U-Cd expressed in micrograms per liter. In the study by Adams et al. (2011), for instance, women had a mean urinary creatinine concentration around 0.4 g/L; thus, U-Cd values were overestimated for a large proportion of their subjects because of very low urinary creatinine values (< 0.3 g/L). The study by Olsson et al. (2002) involved very small groups of former smokers (10 females and 16 males); the males were much older than their referents, so no conclusion can be drawn about their higher U-Cd levels. Results from Paschal et al. (2000) are much more conclusive because they were based on a multivariate analysis of the National Health and Nutrition Examination Survey (NHANES) III database (*n* = 22,162) stratified by never-, current, and former smokers according to serum cotinine. In agreement with our study, these authors observed no increase of U-Cd in former smokers who had even “minimally” lower U-Cd levels than current smokers. Male and female current smokers in that study had mean U-Cd levels higher than that of never-smokers (0.34 and 0.42 µg/L, respectively), which fits rather well with our estimates. Paschal et al. (2000) also provided evidence of coexcretion of Cd and albumin in urine, thus anticipating our observations and those of Akerstrom et al. (2013).

We agree with Adams and Newcomb that there is no better way to assess individual exposure to Cd than by measuring the metal directly in urine or in blood. However, the question is whether one can reliably assess the long-term effects of low-level environmental Cd by means of a biomarker that reflects mostly recent exposure. A cautious interpretation of data is also needed because U-Cd is physiologically linked to proteinuria and albuminuria (Akerstrom et al. 2013), which are well-known predictors of bone and cardiovascular diseases (Barzilay et al. 2013; Smink et al. 2012).
